# Rescue using NOTES during endoscopic ultrasound-guided hepaticogastrostomy, after maldeployment of fully covered self-expandable metal stent

**DOI:** 10.1055/a-2086-2062

**Published:** 2023-05-26

**Authors:** Xiaohua Ye, Xiaoju Su, Ting Yang, Bo Li, Lei Wang

**Affiliations:** 1 Department of Gastroenterology, Affiliated Jinhua Hospital, Zhejiang University School of Medicine, Jinhua, Zhejiang, P. R. China; 2 Department of Gastroenterology, Digestive Endoscopy Center, Changhai Hospital, Naval Medical University, Shanghai, P. R. China

A 56-year-old man was admitted to our hospital with obstructive jaundice caused by extrahepatic cholangiocarcinoma that developed from a congenital choledochal cyst ( Fig. 1 ). Endoscopic retrograde cholangiopancreatography was hampered by ampullary effacement caused by malignant invasion. Endoscopic ultrasound (EUS)-guided hepaticogastrostomy was therefore attempted using an 8-cm fully covered self-expandable metal stent (FCSEMS) (Boston Scientific, Marlborough, Massachusetts, USA).

**Fig. 1:**
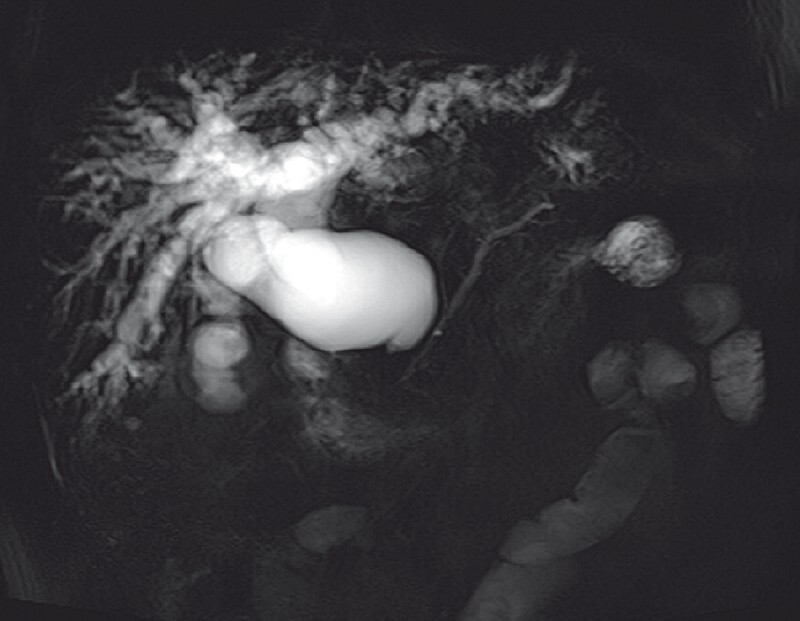
Imaging findings of extrahepatic cholangiocarcinoma developing from a congenital choledochal cyst.

A linear echoendoscope was advanced to the stomach. The intrahepatic bile duct (B3) was punctured with a 19G aspiration needle ( Fig. 2 ). A 0.035-inch guidewire was subsequently inserted via the aspiration needle. Following release of the FCSEMS, cholangiography revealed maldeployment of the proximal flange of the stent into the abdominal cavity.

**Fig. 2:**
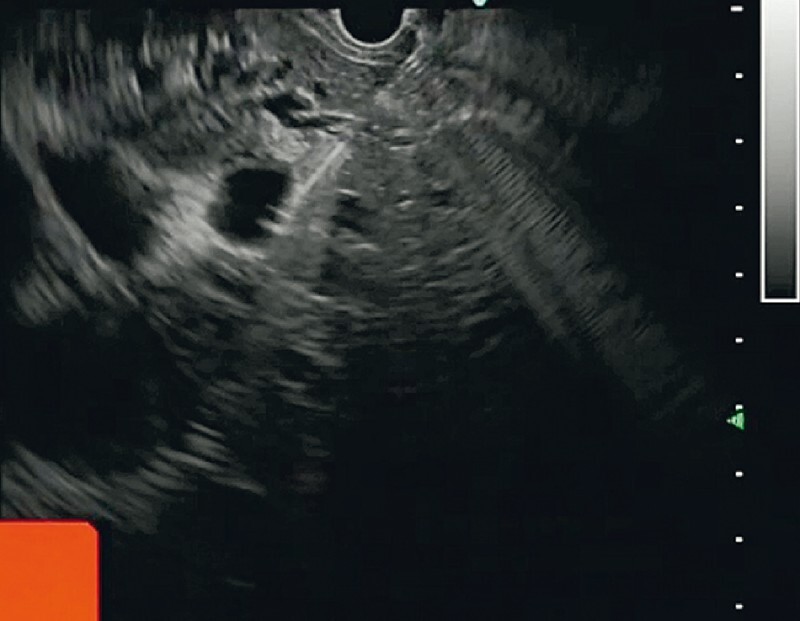
Endoscopic ultrasound (EUS) view of puncture of the dilated intrahepatic bile duct.

We then switched the echoendoscope to a gastroscope ( Fig. 3 ). The entire thickness of the gastric wall was incised using a DualKnife in the puncture location. The gastroscope was further inserted into the abdominal cavity to visualize the position of the FCSEMS. Repositioning of the FCSEMS was achieved by using a foreign-body forceps to pull out the proximal flange through the incision in the gastric wall. Finally, the defect in the gastric wall was sutured with endoclips ( Video 1 ). Cholangiography confirmed that the FCSEMS was in place. The post-procedure period was uneventful and the bilirubin level was improved.

**Fig. 3:**
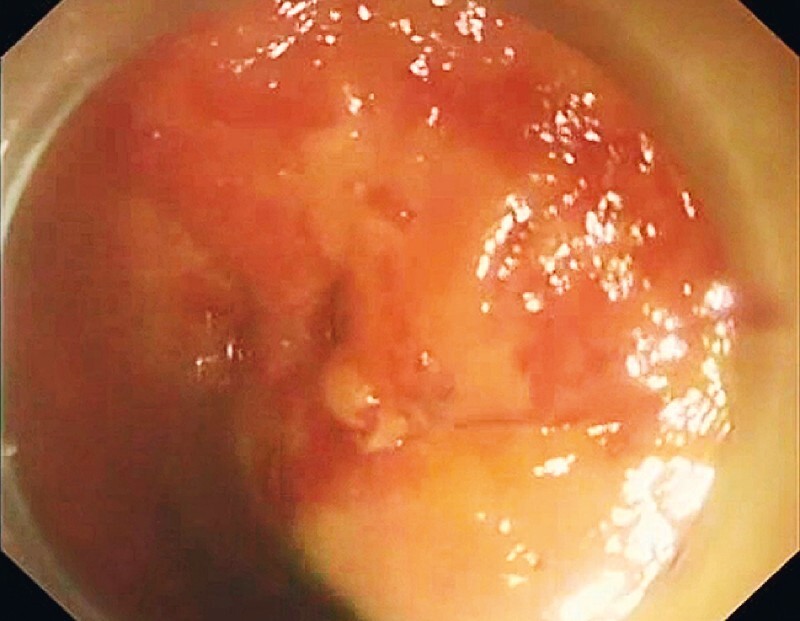
A gastroscope did not detect the proximal flange of the stent in the lumen of the gastroesophageal junction.

**Video 1** Following maldeployment of the proximal flange of the stent into the abdominal cavity during endoscopic ultrasound-guided hepaticogastrostomy, transgastric natural orifice transluminal endoscopic surgery (NOTES) was used as a rescue procedure.


Stent maldeployment during EUS-guided hepaticogastrostomy is a significant adverse event that needs to be immediately managed 1 . The rescue approach described here, which employs transgastric natural orifice transluminal endoscopic surgery (NOTES), avoids the need for emergency surgery, as well as sacrifice of the stent, hence saving on cost.

Endoscopy_UCTN_Code_CPL_1AL_2AC

## Notes

Endoscopy E-Videos

https://eref.thieme.de/e-videos
*E-Videos* is an open access online section of the journal *Endoscopy* , reporting on interesting cases and new techniques in gastroenterological endoscopy. All papers include a high-quality video and are published with a Creative Commons CC-BY license. Endoscopy E-Videos qualify for HINARI discounts and waivers and eligibility is automatically checked during the submission process. We grant 100% waivers to articles whose corresponding authors are based in Group A countries and 50% waivers to those who are based in Group B countries as classified by Research4Life (see: https://www.research4life.org/access/eligibility/ ). This section has its own submission website at https://mc.manuscriptcentral.com/e-videos
